# Comparative palatability of two veterinary dewormers (Milpro^®^ and Milbemax^®^): a blinded randomised crossover cat study

**DOI:** 10.1136/vetreco-2014-000080

**Published:** 2014-12-30

**Authors:** N. Bernachon, D. McGahie, D. Corvaisier, E. Benizeau, N. Crastes, G. Chaix

**Affiliations:** 1 Medical Department, Virbac, Carros, France; 2 Department of R&D, Virbac, Carros, France

## Abstract

**Background:**

The combination of milbemycin oxime–praziquantel is widely used against the most common tapeworms and roundworms affecting cats. New veterinary presentations of this combination have recently been approved.

**Objective:**

The objective of this study was to compare the palatability of two products using this combination, Milpro^®^ and Milbemax^®^.

**Methods:**

In all, 20 adult cats and 20 kittens were offered each product according to a randomisation table using a blinded crossover design. Prehension from the bowl, prehension from the hand and total consumption were assessed.

**Results:**

Both presentations were very well tolerated in adult cats and kittens. Total prehension in adult cats and kittens was 100 and 45 per cent, respectively, for Milpro^®^, and 95 and 30 per cent, respectively, for Milbemax^®^. The percentages of adult cats and kittens which swallowed the pill after taking it into their mouth (total spontaneous consumption) were respectively 40 and 45 per cent for Milpro^®^, and 35 and 20 per cent for Milbemax^®^.

**Conclusion:**

In this study, both presentations were highly attractive to cats and their respective coatings successfully covered the unpleasant odour of praziquantel, which usually repels cats. These results indicate that the palatability of Milpro^®^ is at least as good as Milbemax^®^ and both tablets are well accepted by adult cats and kittens.

## Introduction

Combined dewormers are commonly used in companion animal veterinary practice as they have the advantage of being active against roundworms and tapeworms simultaneously. As these drugs are intended to be given to healthy animals, an excellent safety profile is also important. Milbemycin oxime is a macrocyclic lactone highly effective against a broad spectrum of nematodes and praziquantel is active against the major cestodes of companion animals; both have excellent safety profiles in dogs and cats (Ide and others 1993, Sakamoto 1977, Schenker and others 2005, Di Cesare and others 2014). The combination of these two active ingredients offers a large spectrum of action against the most common worms affecting domestic carnivores. In cats, it is indicated for the treatment of mixed infections by a large variety of immature and adult cestodes, including *Dipylidium caninum* and *Taenia* species and common adult nematodes, such as *Ancylostoma tubaeforme* and *Toxocara cati* (Dey-Hazra 1976, Sakamoto 1977, Thomas and Gönnert 1978, Humbert-Droz and others 2004, Schenker and others 2007, Wolken and others 2012). The milbemycin oxime–praziquantel combination can also be used for the prevention of heartworm disease (*Dirofilaria immitis*), if concomitant treatment against cestodes is indicated (Grieve and Frank 1991, Tagawa and Okano 1993). This combination has also been proven to be efficient on some new emerging zoonoses, such as *Thelazia calipaeda* (Motta and others 2012).

However, efficiency and safety are not the only requirements for a veterinary dewormer, particularly when it is intended to be given to cats. Oral administration of drugs can be very difficult in this species and the reluctance to accept oral administration can be worse if the tablet is too big or if it has an unpleasant smell or taste, leading to a lack of compliance (Thombre 2004, Hautala and others 2014). Praziquantel is well known for its bitterness and its unpleasant odour (Taste masking compositions of Praziquantel 2013). For these reasons, the first veterinary presentations of milbemycin oxime and praziquantel marketed for cats and kittens (Milbemax^®^, Novartis, France) were small flavour-coated tablets. A new veterinary presentation of the combination of milbemycin oxime and praziquantel for cats and kittens (Milpro^®^, Virbac, France) has recently been approved. Given that both Milbemax^®^ and Milpro^®^ cat and kitten presentations are small flavour-coated tablets of similar size, it could be assumed that they would have a similar acceptability rate. Nevertheless, the flavour in the coating is different between the two presentations. Indeed, the coating of Milpro^®^ tablets for cats and kittens contains poultry liver powder, which is a natural flavouring agent, whereas the coating of Milbemax^®^ tablets for cats and kittens contains an artificial beef flavour. This difference could potentially result in different palatability rates. Thus, the objective of the present study was to compare the palatability of these two commercial presentations.

## Materials and methods

Forty healthy entire European cats, living in a closed colony and bred for research, were included in the study. Twenty were adult cats, all previously exposed to praziquantel, aged from 7 to 52 months (weight range 3.35–5.23 kg). The other 20 were kittens aged from 15 to 25 weeks (weight range 1.31–1.96 kg), which have never been exposed to praziquantel. The results for adult cats and kittens were analysed separately.

Two products were tested: Milpro^®^ and Milbemax^®^. These are both small, non-round, flavour-coated tablets containing milbemycin oxime and praziquantel. For both products, the adult presentation contains 16 mg milbemycin oxime and 40 mg praziquantel. The recommended dosage is half-a-tablet for cats between 2 and 4 kg bodyweight and a whole tablet for cats over 4 kg and up to 8 kg bodyweight. Both products are also presented in a kitten presentation containing 4 mg milbemycin oxime and 10 mg praziquantel. The recommended dosage is one tablet for kittens between 1 and 2 kg bodyweight. For the purposes of the study, they were removed from their primary packaging immediately before use and provided to the investigator as simply ‘Product A’ and ‘Product B’. The investigator was not familiar with either product and was blinded as to the identity of the products used in this study.

All animals were housed individually for this study to allow accurate assessment of the palatability and follow the tolerance of the animals for each product. On day 1, the animals were offered either product A or B according to a randomisation table. On day 2, the animals were offered the other product such that each cat tested both products in a crossover design.

The products were offered to the animals at the correct dosage (corresponding to their bodyweight) one hour before feeding in the morning. Initially, the tablets were placed in the empty food bowl for two minutes. In cases where the animal did not spontaneously take the tablet in its mouth from the bowl within this time, it was additionally presented in the hand for 30 seconds. The outcome measures assessed were:

- Prehension (defined as the animal voluntarily taking the tablet in the mouth, whether or not it was subsequently consumed).

Prehension was further split into prehension from the bowl and prehension from the hand.

- Total consumption (the tablet was voluntarily completely swallowed by the animal whether or not it was crunched before swallowing).

If the tablet was crunched and partially rejected, this was classed as refusal, even when the majority was swallowed, due to the need to avoid underdosing for this type of product.

Statistical analysis was performed using SAS V.9.3 software. Groups were compared using a χ^2^ test or a Fisher exact test, with α=5 per cent.

## Results

Both presentations were very well tolerated in adult cats and kittens without any adverse events reported during the study.

**FIG 1:**
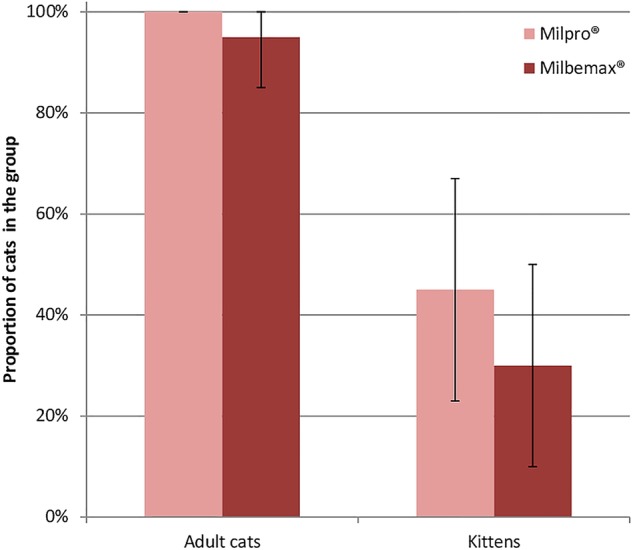
Total prehension rates: proportion of cats taking the tablet into the mouth. Error bars represent the 95% CIs

Total prehension rates in adult cats and kittens for Milbemax^®^ and Milpro^®^ are presented in Fig 1. In adult cats, prehension from the bowl and hand was 90 and 5 per cent, respectively, for Milbemax^®^, and 95 and 5 per cent, respectively, for Milpro^®^. In kittens, prehension from the bowl and hand was 25 and 5 per cent, respectively, for Milbemax^®^, and 40 and 5 per cent, respectively, for Milpro^®^.

**FIG 2:**
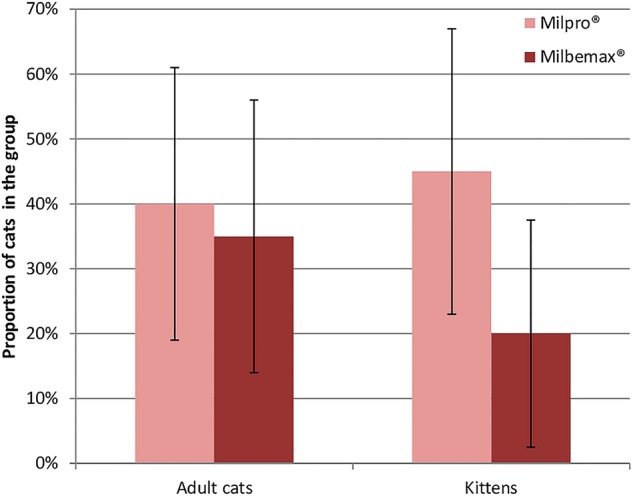
Spontaneous total consumption rates. Error bars represent the 95% CIs

Total spontaneous consumption rates in adult cats and kittens for Milbemax^®^ and Milpro^®^ are shown in Fig 2.

In both adults and kittens, prehension and consumption of Milbemax^®^ and Milpro^®^ were not statistically different, as shown in Table 1.

**TABLE 1:** Number of cats or kittens per category and statistical comparisons

	Milpro^®^	Milbemax^®^	P value
Adult Cat prehension			1.00
Prehension from the bowl	19	19	
Prehension from the hand	1	0	
Total prehension	20	19	
Adult Cat prehension			1.00
Spontaneous total consumption	8	7	
Partial consumption or refusal	12	13	
Kitten prehension			0.33
Prehension from the bowl	8	5	
Prehension from the hand	1	1	
Total prehension	9	6	
Kitten prehension			0.09
Spontaneous total consumption	9	4	
Partial consumption or refusal	11	16	

## Discussion

In this study, both Milpro^®^ and Milbemax^®^ tablets for cats presented excellent total prehension rates in adult cats and acceptable prehension rates for kittens without any statistically significant difference. These results indicate that both tablets are highly attractive to cats and that their respective coatings successfully cover the unpleasant odour of praziquantel, which usually repels cats (Taste masking compositions of Praziquantel 2013). One explanation for the noticeable gap between adult and kitten prehension rates could be found in the great susceptibility of this species to neophobia. This is a natural feeding behaviour defined as the avoidance of a new food compared with the usual food. Previous feeding experience, especially in the peri-natal period, also has an important impact on the willingness of cats to experiment with novel foods in the future. It could thus be hypothesised that the difference seen in this study is because the kittens could have been previously less exposed to a broad panel of flavours than the adult cats. Unfortunately, it was not possible to collect the feeding history of the animals to validate this hypothesis. In any case, this ‘fixation of food habits’, if present, can be overcome by offering the new food every day in familiar and quiet conditions, even if the cat initially does not want to consume it, for at least three days. This persistent exposure to the new food can lead to an acceptation of a new diet (Bradshaw and others 2000, Bourgeois and others 2006, Hepper and others 2012). It could be interesting to assess a similar strategy for the administration of drugs by progressively accustoming kittens to the smell of a new tablet, while taking great care to avoid being in an unfamiliar environment. Indeed, it has been proven that cats may prefer a new diet when they are fed in their normal housing and refuse the same new diet when placed in an unknown situation (Remillard and Crane 2000).

The gap between adult and kitten total spontaneous consumption rates was far smaller than the gap for prehension, with a slight, but statistically non-significant advantage for the Milpro^®^ tablets compared with Milbemax^®^ in both cases. In kittens, the spontaneous consumption was similar to the prehension rate for both products, with the most striking result being that every kitten which took a Milpro^®^ tablet in the mouth ingested it completely. As in kittens, the total spontaneous consumption was assessed as acceptable for a praziquantel-containing tablet, in the adult cats group, with 40 per cent for Milpro^®^ and 35 per cent for Milbemax^®^. The difference between prehension and total consumption in adult cats could be explained by exposure to the very bitter taste of praziquantel if the coated tablet is crunched, which happens more frequently in adults, while young animals are more inclined to swallow the entire tablet once it is accepted (Taste masking compositions of Praziquantel 2013). The coating of the tablet certainly prevents the cat from tasting the praziquantel, and thus if the tablet was not chewed the cat would be still receptive to receiving a second dose in the future. As not all animals voluntarily consume the entire tablet, direct administration into the animal's mouth by the owner will still be required in these cases in order to ensure correct dosing. In such cases, the small size of the tablets combined with the attractive smell (as evidenced by the excellent rate of prehension demonstrated in this study) will facilitate the direct administration of the product.

In this study, the animals were similar in both groups in order to assure equal comparison between the two products. All adult cats which were included were experimental cats under 52 months of age. This may represent a potential limitation of this study as a lot of cats that will be given a veterinary dewormer in the field could be older cats as well. Sometimes when cats become older, their food acceptance can decrease due to a variety of factors. Nevertheless, the comparison between groups remains legitimate.

The results reported here indicate that the palatability of Milpro^®^, a new small flavour-coated tablet for cats and kittens, which combines milbemycin oxime and praziquantel, is at least as good as Milbemax^®^, the first veterinary combination of milbemycin oxime and praziquantel marketed for cats and kittens. They also confirm that both presentations are well accepted by cats and kittens.
